# Beyond FOXP3: a 20-year journey unravelling human regulatory T-cell heterogeneity

**DOI:** 10.3389/fimmu.2023.1321228

**Published:** 2024-01-12

**Authors:** Samikshya Santosh Nirmala, Kayani Kayani, Mateusz Gliwiński, Yueyuan Hu, Dorota Iwaszkiewicz-Grześ, Magdalena Piotrowska-Mieczkowska, Justyna Sakowska, Martyna Tomaszewicz, José Manuel Marín Morales, Kavitha Lakshmi, Natalia Maria Marek-Trzonkowska, Piotr Trzonkowski, Ye Htun Oo, Anke Fuchs

**Affiliations:** ^1^ Center for Regenerative Therapies Dresden, Technical University Dresden, Dresden, Germany; ^2^ Centre for Liver and Gastrointestinal Research and National Institute for Health Research (NIHR) Birmingham Biomedical Research Centre, Institute of Immunology and Immunotherapy, University of Birmingham, Birmingham, United Kingdom; ^3^ Department of Academic Surgery, Queen Elizabeth Hospital, University of Birmingham, Birmingham, United Kingdom; ^4^ Department of Renal Surgery, Queen Elizabeth Hospital Birmingham, University Hospitals Birmingham NHS Foundation Trust, Birmingham, United Kingdom; ^5^ Department of Medical Immunology, Medical University of Gdańsk, Gdańsk, Poland; ^6^ International Centre for Cancer Vaccine Science, University of Gdańsk, Gdańsk, Poland; ^7^ Liver Transplant and Hepatobiliary Unit, University Hospitals Birmingham NHS Foundation Trust, Birmingham, United Kingdom; ^8^ Birmingham Advanced Cellular Therapy Facility, University of Birmingham, Birmingham, United Kingdom; ^9^ Centre for Rare Diseases, European Reference Network - Rare Liver Centre, Birmingham, United Kingdom

**Keywords:** regulatory T cells, FOXP3, Treg markers, Treg heterogeneity, Treg function, Treg therapy, Treg chemokine receptors

## Abstract

The initial idea of a distinct group of T-cells responsible for suppressing immune responses was first postulated half a century ago. However, it is only in the last three decades that we have identified what we now term regulatory T-cells (Tregs), and subsequently elucidated and crystallized our understanding of them. Human Tregs have emerged as essential to immune tolerance and the prevention of autoimmune diseases and are typically contemporaneously characterized by their CD3^+^CD4^+^CD25^high^ CD127^low^FOXP3^+^ phenotype. It is important to note that FOXP3^+^ Tregs exhibit substantial diversity in their origin, phenotypic characteristics, and function. Identifying reliable markers is crucial to the accurate identification, quantification, and assessment of Tregs in health and disease, as well as the enrichment and expansion of viable cells for adoptive cell therapy. In our comprehensive review, we address the contributions of various markers identified in the last two decades since the master transcriptional factor FOXP3 was identified in establishing and enriching purity, lineage stability, tissue homing and suppressive proficiency in CD4^+^ Tregs. Additionally, our review delves into recent breakthroughs in innovative Treg-based therapies, underscoring the significance of distinct markers in their therapeutic utilization. Understanding Treg subsets holds the key to effectively harnessing human Tregs for immunotherapeutic approaches.

## 1 Introduction

FOXP3^+^ regulatory T-cells (Tregs) represent 4-7% of the CD4^+^ T-cell population and are essential for regulating peripheral tolerance and immune homeostasis. Early phase clinical trials have demonstrated proof-of-principle for the use of isolated and expanded polyclonal Tregs in the treatment of inflammatory disorders, transplant rejection and autoimmune diseases. Treg research traces its origins back more than 40 years. The first evidence for thymus-derived cells with suppressive function came from mouse thymectomy experiments conducted in the early 1970s (1). Mice thymectomised between day two and four of life developed severe autoimmunity, which did not occur if thymectomy was performed before 24 hours or after day five of life (2). This led to the hypothesis that autoreactive T-cells are exported from the thymus within the first few days of life, followed later by an anergising subset of T-cells. Importantly, autoimmunity could be reversed with thymic transplantation at day ten. In line with these findings, Gershon and Kondo showed thymus-derived lymphocytes were crucial for inducing tolerance (3), and tolerance could be adoptively transferred to naïve recipients (4). The presence of suppressor T-cells, however, was questioned when several other groups failed to verify the mechanisms of suppression postulated using the novel techniques, including monoclonal antibodies and Sanger sequencing. These negative findings together with a lack of specific markers led to a loss of interest in the suppressor T-cell theory in the mid-1980s.

In the 1990s, a significant breakthrough emerged with the identification of regulatory T-cells (Tregs), by Sakaguchi and colleagues following the work on tolerance induction by Hall (5–7). Sakaguchi et al. demonstrated that Tregs could be distinguished by the expression of the IL-2 receptor α-chain, CD25, which was exclusive to a minority of CD4^+^ cells, facilitating the isolation of relatively pure Tregs populations from mice. The Shevach group provided the first direct evidence of Tregs inhibiting CD4^+^ effector T-cell proliferation in culture (8). In 2001, several research groups independently succeeded in identifying Tregs in human peripheral blood utilizing CD25 (9–11). Given human conventional T-cells upregulate CD25 upon activation, it soon became evident that this early cell-surface marker had certain limitations, despite David Hafler’s group demonstrating CD25 expression directly correlates with suppressive capacity (12).

The discovery of Foxp3, a forkhead box transcription factor, as the master control gene in mouse Treg development and function, provided further progress in the field and crucial insights into Treg regulatory mechanisms (13–15). It also explained the fatal lymphoproliferative disease observed in scurfy mice, which carry a frameshift mutation resulting in scurfin lacking the forkhead domain, and consequently autoimmunity (16). Intranuclear FOXP3 expression was demonstrated in human Tregs in 2005 (17), and mutations in FOXP3 explained the immune dysregulation, polyendocrinopathy, enteropathy and X-linked clinical syndrome (IPEX) (18, 19). Due to its intranuclear localization necessitating cell fixation, this new marker could not be used for the isolation of live Tregs. The discovery that FOXP3 expression could be induced in conventional human T-cells upon activation further complicated the identification of pure human Tregs (20).

In 2006, the expression of the IL-7 receptor α-chain CD127 was found to inversely correlate with FOXP3 expression and suppressive function (21, 22). In combination with CD25, low CD127 expression levels facilitated the isolation of live human Tregs with high purity and FOXP3 expression in post-sort analyses. Shortly thereafter, a more specific method to determine Treg purity among FOXP3 expressing cells was identified. Methylation analysis of the FOXP3 conserved non-coding sequence 2 (CNS2) (also termed the Tregs-Specific Demethylated Region (TSDR)) is at least partially methylated in conventional T-cells, but is completely demethylated in *bona fides* FOXP3^+^ Tregs and remains the most specific method of identifying Tregs (23, 24).

**Figure 1:**
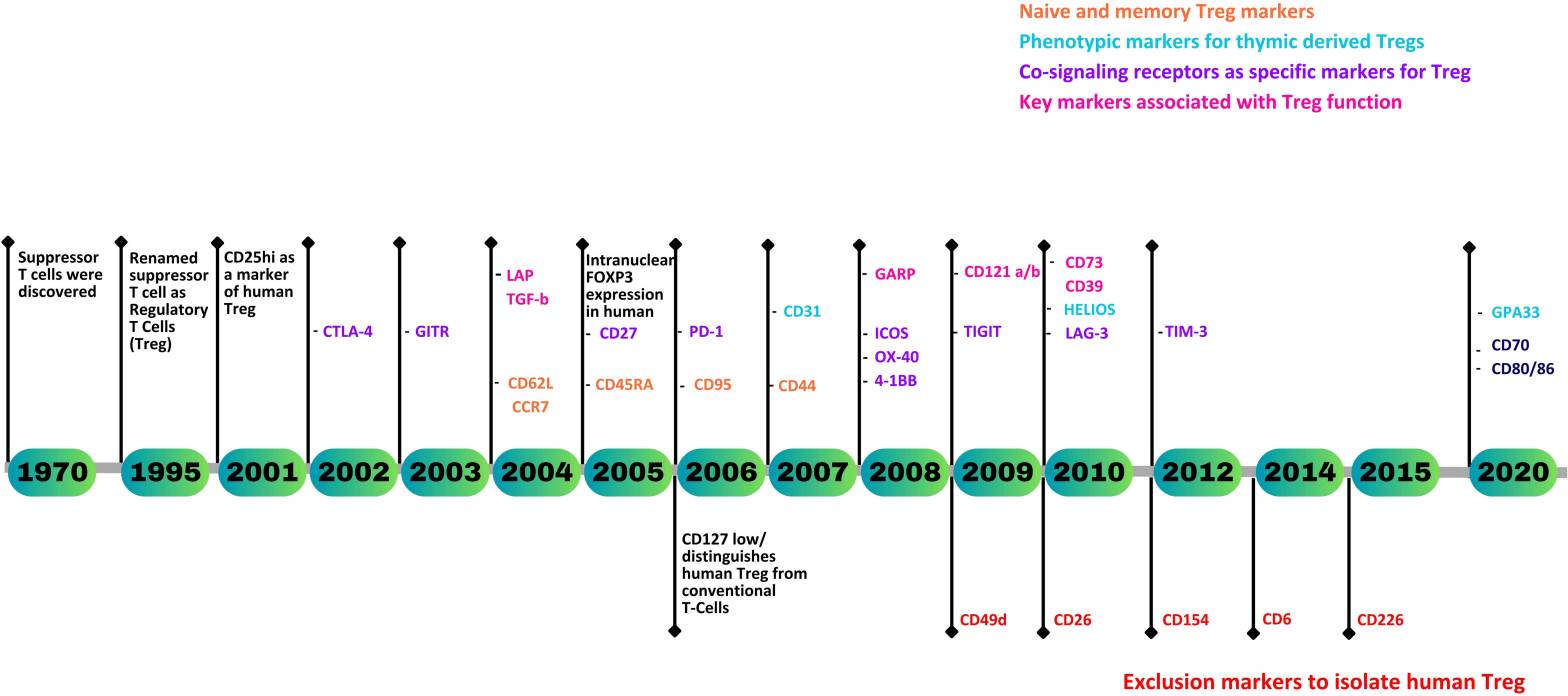
A timeline representing the discovery of human Treg markers.

Currently, CD3^+^CD4^+^CD25^hi^CD127^lo^ remains the most widely used gating strategy for the isolation of viable Tregs when cell sorting, with intranuclear staining of FOXP3 used to confirm purity and function. Of note, however, FOXP3, whilst exclusive to Tregs in mice, does not provide unambiguous identification of human Tregs. Finding a specific marker for human Tregs remains an unmet need. Nevertheless, over the past two decades, a substantial number of novel Treg markers associated with their origin, maturity, stability, and function have been identified (**Figure 1**). This review gives a comprehensive analysis of human Treg markers, facilitating the precise identification of CD4^+^ Tregs and their subsets, enabling the characterization of Tregs in a variety of immunological disorders and physiological processes.

## 2 Naïve and memory T-cell components as markers of Treg heterogeneity and functional diversity

Until 2005, Tregs in the peripheral blood were believed to predominantly exhibit a memory-like phenotype, characterized by the expression of CD45RO, until Valmori et al. described the existence of circulating Tregs expressing the naïve T-cell marker CD45RA (25). The prevalence of this subset inversely correlates with age, and concomitantly falls with the naïve CD4^+^ T-cell fraction, whilst the total Treg frequency remains constant throughout life. Naïve Tregs also have longer telomeres than their memory counterparts, similar to naïve conventional CD4^+^ T-cells. These naïve Tregs were postulated to be derived from CD4^+^CD25^+^ cells, selected in the thymus as precursors to antigen-experienced memory Treg subsets, awaiting TCR stimulation-mediated maturation, leading to an interest in umbilical cord blood Tregs.

A comparative study of CD45RA^+^ naïve Tregs from umbilical cord blood and adult blood found slightly lower levels of CD25 and FOXP3 expression in both when compared to memory Tregs, but similar *in vitro* suppressive capacities to memory Tregs (26). In 2006, Hofmann and Edinger showed the CD45RA^+^ naïve fraction of adult Tregs, which phenotypically resemble cord blood Tregs, gives rise to a more homogeneous Treg pool upon *in vitro* expansion as compared to bulk Tregs, thus proposing naïve Tregs as the optimal source of Tregs for adoptive cell therapies (27). They also demonstrated the superior stability of CD45RA^+^FOXP3^+^ cells. However, after three weeks of *in vitro* expansion with repetitive stimulation, CD45RA^−^ Tregs preferentially down-regulated FOXP3 expression and were liable to produce pro-inflammatory cytokines (28). Miyara et al. confirmed the enhanced proliferative capacity of naïve Tregs and found that once activated, naïve Tregs upregulate FOXP3 and convert to an activated memory phenotype, all whilst exerting suppression during and after their proliferation and conversion (24).

Furthermore, both adult naïve and cord blood Tregs were shown to express significant levels of the secondary lymph node homing receptors CD62L (L-selectin) and CCR7 (25, 26). These markers, along with CD45RA/RO are commonly used to distinguish between naïve (CD45RA^+^/RO^-^, CD62L^hi^, CCR7^hi^), effector memory (Tem) (CD45RA^-^/RO^+^, CD62L^lo^, CCR7^lo^), and central memory (Tcm) (CD45RA^-^/RO^+^, CD62L^hi^, CCR7^hi^) conventional CD4^+^ T-cells. While studies have identified CCR7 as a differential marker defining Tem and Tcm subsets within the human CD4^+^CD25^+^ Treg population (29, 30), there is limited evidence for clear Tem and Tcm characteristics in Tregs based solely on these markers. Nevertheless, the expression of CD62L and CCR7 is crucial to the migration of Tregs to secondary lymphoid organs in murine models, proving essential to Treg-mediated suppression of autoimmune (30–33), allograft and other immune responses (34–38). Interestingly, even after undergoing extensive *in vitro* polyclonal expansion, human CD4^+^CD25^high^ Tregs retain the expression of CD62L and CCR7 (39). It would be intriguing to monitor CD62L and CCR7 expression in Tregs used for human cell therapy trials, examining if, and how, these markers change with Treg migration to secondary lymphoid organs and inflamed tissues.

In contrast to conventional CD4^+^ T-cells, the majority of Tregs in adult peripheral blood exhibit elevated levels of CD45RO and CD95, making them susceptible to CD95L-mediated apoptosis (40). Interestingly, CD4^+^CD25^+^FOXP3^+^ T-cells present in cord blood are predominantly naïve, display low expression of CD95 like adult naïve Tregs, and remain resistant to FAS-mediated apoptosis. However, upon brief stimulation with anti-CD3/CD28 monoclonal antibodies, cord blood Tregs significantly upregulate CD95, sensitizing them to CD95L (40).

The expression of CD44, a cell surface molecule with important roles in activation, migration, and apoptosis, is linked to FOXP3 expression and Treg function in mice (41). Similar studies in human Tregs indicated that CD44 enhances the suppressive action of CD4^+^CD25^+^ Tregs (42). Further investigation in 2009 demonstrated that CD44 co-stimulation plays a crucial role in enhancing the persistence and function of FOXP3^+^ Tregs through the production of IL-2, IL-10, and TGF-β1 in both humans and mice (43).

## 3 Phenotypic markers of Tregs derived from the thymus

It was formerly believed that natural Tregs are thymically derived CD4^+^ cells that constitutively display CD25. Work in mice demonstrating the capacity for naïve CD4^+^ T-cells to transform into FOXP3^+^ Tregs in the periphery led to a paradigm shift. Subsequent categorization of Treg subsets now includes thymically derived Tregs (tTregs) and peripherally induced Tregs (pTregs) (44). However, the presence of pTregs in humans remains a controversial topic. Multiple reports have indicated that pTregs are plastic and can produce inflammatory cytokines (45–47). Significant attention has been devoted to identifying phenotypic markers capable of distinguishing between pTregs and tTregs.

Helios (Ikzf2) is a member of the Ikaros transcription factor family and is restricted to T lymphocytes. Microarray studies characterizing unique gene signatures in FOXP3+ mouse Tregs provided the first indication of Helios as a Treg-specific gene (48, 49). In 2010, the Shevach group published work indicating Helios to be a potential marker specific to FOXP3^+^ tTregs in mice and humans (50). Approximately 70% of Tregs in human peripheral blood and mouse peripheral lymphoid tissues, as well as over 95% of Tregs in mouse thymi, were Helios positive. Additionally, Helios expression was not seen neither in murine nor human *in vitro* or *in vivo* induced Tregs. Interestingly, the majority of FOXP3^+^ Tregs producing IL2, IL17, and IFN-γ belong to the Helios negative subset. Additionally, more than 90% of Tregs in human cord blood and thymic samples express Helios (51). Crucially, the Helios-negative Treg subset, comprising less than 10% of the total, does not originate exclusively from the thymus. Studies have suggested, in the case of cord blood, Helios-negative Tregs are generated in the periphery during fetal gestation, while those found in the thymus are recirculated Tregs from the periphery. Subsequent studies have demonstrated that human FOXP3^+^Helios^+^ Tregs demonstrate less than 10% CpG methylation in the TSDR, while FOXP3^+^Helios^−^ Tregs are more than 40% methylated (52, 53). Thus, the early consensus was Helios could serve as a marker for tTregs, with Tregs lacking Helios representing pTregs.

More recent studies have disputed this consensus (54, 55), demonstrating inducible Helios expression in pTregs (56), findig Helios expression with Treg activation and proliferation (57). The coexistence of both Helios positive and negative human FOXP3^+^ tTregs has also been demonstrated (58), with further studies supporting the notion that Helios expression cannot differentiate between tTregs or pTregs (59). Thus, the question of whether Helios is a reliable specific marker for tTregs remains open (60).

A 2007 study by Hass et al. on Treg dysfunction in multiple sclerosis demonstrated a significant correlation between Treg-mediated immunosuppression and the presence of recent thymic emigrants (RTE) Tregs (61). Naïve Tregs expressing CD31 (PECAM1) play a role in the functional characteristics of the entire Treg population. CD45RA^+^CD45RO^−^CD31^+^ naive Tregs have been observed to decline with age in healthy individuals, contrasting with their CD31^−^ memory Tregs counterparts. Subsequent studies indicate that, unlike memory Tregs, a substantial proportion of CD45RA^+^ naive Tregs express CD31 (62, 63).

A novel marker, Neuropilin-1 (Nrp-1), was identified in 2012 by Bluestone and Lafaille. It was found to be expressed on tTregs, but not on *in vivo* or *in vitro* induced pTregs in mice (64, 65). While these murine studies are useful to our understanding of Treg development and putative tTreg biomarkers, the translation of these findings into the human context remains disputed (59, 66, 67).

Glycoprotein A33 (GPA33) is a cell surface antigen and a member of the immunoglobulin superfamily. While this marker is known to be expressed in approximately >95% of primary and metastatic colon cancers, its presence on normal tissues and cells was previously unreported (68). In a 2020 study, Opstelten et al. identified GPA33 on tTregs, reporting high levels of mRNA and surface protein expression in CD4^+^CD25^+^CD45RA^+^ naive Tregs (69). All CD4^+^CD25^+^CD127^-^GPA33^+^ Tregs were shown to express FOXP3 and Helios, whereas only 86% of CD4^+^CD25^+^CD127^-^CD45RA^+^ expressed FOXP3 and Helios, suggesting CD127^-^GPA33^+^ selects naïve Tregs with enhanced purity, lineage stability, and suppressive function. Subsequent single-cell transcriptomic studies have demonstrated that GPA33 is acquired by pre-Tregs in a late developmental stage, prior to the acquisition of CD45RA, validating GPA33 as a tTreg marker (70). Combined, this suggests GPA33^+^ is found on a highly pure and stable population of CD45RA^+^ Tregs originating from the thymus. It will be interesting to assess the applicability of this intriguing new marker for the isolation and expansion of functional Tregs, especially as the function of GPA33 is presently unclear.

## 4 Co-signaling receptors as markers of Treg proliferation and function

Co-stimulatory and co-inhibitory receptors, collectively described as co-signaling receptors, are crucial to regulating the expansion and function of conventional CD4^+^ T-cells, modulating immune responses. Their discovery marked a turning point in our understanding of Treg function, eventually leading to various clinically employed therapeutic agents, called checkpoint inhibitors, which target Co-signaling receptors to restore desired immune responses (71, 72).

Cytotoxic T-lymphocyte–associated antigen 4 (CTLA-4), also known as CD152, is an immunoglobulin superfamily (Ig SF) member expressed by activated inhibitory conventional T-cells (73). CTLA-4 is often referred to as a “moving target” due to its rapid internalization and recycling to the plasma membrane of T-cells (74). Notably, while CTLA-4 is expressed in conventional T-cells upon activation, it is constitutively expressed in resting Tregs, representing one of the hallmarks of this cell subset (75, 76). Post TCR-mediated activation, human Tregs strongly upregulate CTLA-4.

In 2000, work by the Powrie and Sakaguchi groups provided compelling evidence of CTLA-4’s crucial role in contact-dependent suppressive function in mice (76, 77). Subsequent human Treg *in vitro* studies demonstrated conflicting results. Some supported the notion that blocking CTLA-4 using anti-CTLA-4 monoclonal antibodies (mAbs) or Fab fragments reversed the suppressive effect of CD4^+^CD25^+^ Tregs on Teff proliferation (78–80). Other studies came to the opposite conclusion (9, 10, 81, 82), highlighting the complex nature of CTLA-4’s role in Treg-mediated suppression. However, variations in assay conditions, including the type of antigen-presenting cells (APCs) used and the strength of the TCR stimulus may account for the differing results observed. CTLA-4 interacts with CD80/CD86 on APCs with higher affinity and avidity than its counterpart CD28, which competes to interact with CD80/86. As a target gene of FOXP3, CTLA-4 plays a pivotal role in regulating Treg homeostasis by acting as an intrinsic regulator of Treg proliferation. Deletion or blockade of CTLA-4 enhances Treg proliferation and affects the number and function of memory Tregs (83–86). Although the role of CTLA-4 in Treg biology has remained controversial for several years, its established use as a diagnostic marker and therapeutic target for various human pathologies confirms its critical role in Treg-mediated immunosuppression (72, 87–89).

In parallel to CTLA-4, programmed cell death 1 (PD-1) is another co-inhibitory molecule from the Ig SF found in Tregs. In 2003, it was found that blocking the interaction between PD-1 and its ligand, PD-L1, influences Treg suppressive function (82). A more comprehensive 2006 study demonstrated that freshly isolated murine and human Tregs retain PD-1 in the intracellular compartment (90). PD-1 appears to transmigrate to the cell surface upon TCR stimulation, indicating that purifying CD25^+^PD-1^−^ T-cells may give a pure population of resting Tregs, prompting evaluation of PD-1 as a marker to distinguish Tregs from activated CD4+CD25+ T-cells. Francisco et al. showed that the expression of PD-1 minimally converts naive CD4^+^ T-cells into induced Tregs (iTregs) by promoting FOXP3 expression, enhancing iTreg suppressive activity (91). Our current understanding of PD-1/PD-L1 signaling in Tregs is complex and context-dependent, with effects including Treg expansion (92), survival and functionality (93) in addition to suppression of effector T-cells and autoreactive B-cells (94). These effects have implications for tumor immunity and autoimmunity, as reviewed by Cai et al. (95) and the clinical use of PD-1 inhibitors highlights the therapeutic value of targeting this receptor. Nevertheless, further research deciphering the intricate mechanisms of PD-1 signaling in Treg development and function is required.

Inducible T-cell costimulator (ICOS) is another CD28-related member of the immunoglobulin superfamily of molecules (*IgSF*) that stands apart from CTLA-4 and PD-1 by functioning as a costimulatory receptor. ICOS’s discovery on the surface of T-cells following TCR-mediated stimulation in 1999 represented a breakthrough in T-cell research (96). ICOS^+^ T-cells, although a minority among peripheral blood CD4^+^ T-cells, comprise approximately 20% of FOXP3^+^ Tregs (97). Ito et al. observed that both ICOS^+^ and ICOS^–^ Tregs exist in cord blood and neonatal thymi (98) and that ICOS^+^ Tregs are able to produce IL-10 and suppress CD86 upregulation on DCs, while ICOS^-^ Tregs produce TGFβ. Early investigations unveiled a link between elevated ICOS expression on CD4^+^ T-cells and IL-10 production in mice (99). This was later confirmed in humans (98) and demonstrated the central role ICOS has in the differentiation and function of FOXP3^+^ Tregs. Over the past decade, ICOS’s multifaceted roles, including involvement in the production, proliferation and survival of Tregs and enhancing Treg suppressive function have been unraveled (100–103).

CTLA4, PD1 and ICOS are now recognized targets in cancer immunotherapy, leading to an interest in novel Ig SF co-inhibitory receptors such as Lymphocyte activation gene-3 (Lag-3), T-cell (or transmembrane) immunoglobulin and mucin domain 3 (Tim-3), and T-cell immunoreceptor with Ig and ITIM domains (TIGIT), to address CTLA-4 and PD-1 checkpoint inhibitor non-responders.

Lag-3, also called CD223, belongs to the CD2/Signaling Lymphocytic Activation Molecule (SLAM) family of the Ig SF. It was initially discovered as a molecule upregulated on activated CD4^+^ and CD8^+^ T-cells (104) and has subsequently been reported to be highly expressed in both tTregs and pTregs upon activation (105). Lag-3 blockade on Tregs abolishes Treg suppressor function, while ectopic expression of Lag-3 in non-Treg CD4^+^ T-cells confers suppressive activity. Additionally, Lag-3 is necessary for Treg-mediated control of T-cell homeostasis (106). In subsequent years, Lag-3 has emerged as a useful Treg surface marker that can be cleaved into a soluble form (107, 108). Functional studies of Lag-3 in human Tregs report only small proportions of Tregs are LAG-3 positive, although this fraction increases in inflamed tissues such as in the lamina propria of ulcerative colitis (UC) patients, and various tumors (109–111). Thus, Lag-3 may serve as a diagnostic and treatment response biomarker in the future, however further research into the mechanism of action of Lag-3 in Tregs and its impact on T-cells is warranted.

TIGIT, a member of the poliovirus receptor (PVR)-like family of Ig SFs, is a co-inhibitory receptor expressed on many lymphocyte subsets. It is highly expressed on Tregs, where it promotes suppressive function. The first link between TIGIT and human Treg biology was reported in 2009 (112), however, its functional role in Tregs was not shown until 2014 (113). TIGIT is present on the surface of both human and murine Tregs, including tTregs and pTregs. TIGIT-positive Tregs exhibit increased expression of key Treg-associated genes, including FOXP3, CD25, and CTLA-4, and demonstrate enhanced demethylation in the TSDR, indicating lineage stability. Activation of TIGIT on Tregs triggers the production of suppressive molecules like IL-10 and Fgl2. Moreover, TIGIT expression defines a Treg subset that demonstrates selective suppression of Th1 and Th17 but not Th2 responses. Additional studies have reported TIGIT to identify highly suppressive Tregs, indicating the therapeutic potential of TIGIT Tregs in treating disease (114–116).

Tim-3 (HAVCR2) has attracted significant attention as a potential negative regulator of T-cell responses (117, 118). Tim-3 expression is limited to a small subset (2-5%) of Tregs in the periphery during steady-state conditions. However, during immune responses, Tim-3 is upregulated on Tregs, with elevated expression in Tregs infiltrating allografts, tissues, and tumors in mice (119). Subsequent studies have found that Tim-3 is predominantly expressed on tumor-infiltrating Tregs in both human and experimental tumor models (120–126), and Tim-3^+^ Tregs exhibit enhanced suppressive capacity in *in vitro* suppression assays compared to Tim-3^-^ Tregs (122, 124). Moreover, Tim-3^+^ Tregs display increased expression of suppressive molecules (CTLA-4, Lag-3, and PD-1), and enhanced secretion of immunosuppressive cytokines (IL-10 and TGF-β). These, and more recent findings, strongly argue for Tim-3^+^ Tregs cells as promising therapeutic target in cancer immunotherapy (127). Furthermore, a substantial increase in Tim-3 levels are seen following the *ex vivo* expansion of clinical-grade human Tregs, increasing to 29% (128). Sorted Tim-3^+^ Treg populations show significant enhancements in IL-10 and Granzyme B production, indicating the suppressive capacity of this subset and its potential as a cell-based therapy to induce allograft tolerance.

The TNF receptor superfamily also includes several co-stimulatory molecules that have been identified on Tregs, including GITR, 4-1BB, OX40 and CD27.

Glucocorticoid-induced tumor necrosis factor receptor-related protein (GITR), also known as TNFRSF18 and CD357, acts as a costimulatory molecule. While expressed at low levels on naïve T-cells, Tregs and activated T-cells show high surface expression of GITR (129, 130). Soon after the discovery of GITR on murine Tregs (131, 132), studies reported GITR to be co-expressed with CD25 and FOXP3 in human tTregs (133–135). GITR is now an established Treg marker with tTreg specificity, whose gene loci is demethylated in Tregs (136). Tregs upregulate GITR expression upon activation, with levels correlating with Treg immunosuppressive function, leading to the proposal of using GITR as a marker for selecting highly functional Tregs. Additionally, Tregs residence in the tumor microenvironment demonstrates GITR upregulation and GITR’s expression in Tregs has been extensively studied in autoimmune diseases, demonstrating its potential as an immunotherapy target having already shown promise in murine models (137, 138).

4-1BB (also known as CD137), was initially considered a proliferation and activation marker of CD8^+^ conventional T-cells, but was later found to be constitutively expressed in murine CD4^+^CD25^+^ Tregs in two separate DNA microarray studies (132, 139). 4-1BB expression on resting Tregs was subsequently identified, with upregulation demonstrated following 2 days of *in vitro* combined CD3 and IL-2 stimulation (140). Subsequently, in both *in vitro* and *in vivo* conditions, 4-1BB mediated enhanced Treg survival and proliferation was observed, without alteration to immunosuppressive function (140, 141). Additionally, animal studies have shown infusions of agonistic anti–4-1BB mAbs lead to increases in splenic Tregs, with suppression of chemically induced colitis (142). Furthermore, artificial APCs (aAPCs) expressing 4-1BBL have been shown to expand human umbilical cord blood Tregs and enhance their suppressive function (143). Most significantly, it has been found that gating on 4-1BB^+^CD40L^-^ (CD137^+^CD154^-^) identifies both *ex vivo*, and *in vitro* activated Tregs, facilitating the isolation of epigenetically stable antigen-activated Tregs, enabling their rapid functional testing (144, 145).

Along with the discovery of 4-1BB on Tregs, the aforementioned DNA microarray studies also identified OX40 (CD134) to be highly expressed on Tregs, which had previously been recognized as a costimulatory molecule on activated CD4^+^ effector T-cells (132, 139). OX40 signaling is crucial to the development, homeostasis, and suppressive activity of mouse Tregs (146). Early studies of human cord blood Tregs demonstrated significantly higher Treg expansion using aAPCs modified to express OX40L compared to bead- or non-modified aAPCs (143). However, OX40 signaling has also shown the potential to oppose Treg-mediated suppression in antigen-engaged naive T-cells in *in vivo* mouse models (146). The contrasting functions of OX40 in Tregs, which has also been reported in a variety of human and mouse studies, make its exact role in Tregs unclear (147–151). While uncertainty continues regarding OX40 in Tregs, it remains recognized as a Treg activation marker and is believed to contribute to Treg proliferation.

CD27 is present on the majority of CD4^+^ T-cells, interacting with CD70 on APCs, and *in vitro* T-cell expansion can be enhanced using anti-CD27 antibodies (152). Initially discovered through DNA microarray studies (132), CD27 is now recognized for its crucial role in Treg function. Along with CD25, CD27 can identify highly suppressive FOXP3^+^ Tregs (153) and subsequent investigations have illustrated a novel use of CD27 to induce, expand, and select highly suppressive, Ag-specific human Treg subsets (154). Moreover, following the *ex vivo* expansion of human pTregs, CD27 can discriminate between regulatory and non-regulatory cells (155). With multiple studies highlighting CD27 as a reliable marker for the identification of highly functional Tregs (156, 157), CD27 demonstrates significant potential as a marker for the isolation of suppressive Tregs for clinical application.

Potential markers of Tregs exhaustion: Whilst the effects of chronic T-cell stimulation have been extensively studied, our understanding of Treg exhaustion is limited. In conventional T-cells, exhaustion from chronic antigen exposure is characterized by the expression of inhibitory receptors (PD-1, TIM-3, and LAG-3), reduced proliferation, cytokine production, and increased apoptosis (158–161). Studying Treg exhaustion *in vitro* is hampered by the contradictory roles of inhibitory receptors in Tregs, as discussed above. Tonic-signaling chimeric antigen receptors (TS-CARs) have facilitated the first comprehensive investigation of exhaustion in Tregs, revealing that Tregs rapidly acquire an exhaustion-like phenotype, with increased expression of inhibitory receptors and transcription factors including PD-1, TIM3, TOX and BLIMP1, akin to conventional T-cells (162). Epigenetic changes can also be observed with repetitive TCR-mediated stimulation, including those that may affect Treg functionality and lead to exhaustion, with TIM-3 and TIGIT implicated, among others (163). However, further clarification is needed on the implications of these recent studies on these markers for identifying exhausted Tregs.

## 5 Exclusion markers for human Treg isolation

One of the major obstacles hampering the clinical application of Tregs is the lack of suitable extracellular markers, complicating their identification and isolation. Due to a lack of clinical-grade CD4 negative isolation reagents, clinical grade GMP magnetic enrichment typically involves CD8 depletion, an optional CD19 depletion step, followed by CD25 enrichment. Where cell sorting is possible, Tregs are typically isolated as CD4^+^CD25^+/hi^CD127^-/low^. Over the past decade, numerous studies have attempted to identify additional negative selection markers to facilitate the efficient sorting of pure and highly functional Tregs, while eliminating contaminating effector T-cells. In this section, we explore cell surface markers that are specifically absent on Tregs and consider negative selection strategies for Treg isolation for both functional research and clinical application.

CD49d, the α chain of the integrin VLA-4 (α4β1), functions as a costimulatory and adhesion molecule for lymphocyte transmigration into inflamed tissues (164, 165). In 2009, Kleinewietfeld et al. demonstrated CD49d expression on more than 80% of human PBMCs, with expression predominantly on pro-inflammatory effector cells, including non-regulatory CD4^+^CD25^low^ T-cells, cytokine-secreting CD4^+^ effector cells, and FOXP3 expressing Th1- and Th17-like cells. Significantly, approximately 70% of immunosuppressive FOXP3^+^ Tregs are CD49d^-/low^. Considering the differential distribution of CD49d, this marker has been proposed to effectively deplete cells that otherwise contaminate CD25^+^-based Treg preparations (166). This has led to novel CD49d^-^ gating strategies, designed to obtain “untouched” FOXP3^+^ Tregs (i.e. cells that have not been targeted by an antibody during purification) in combination with CD127^-^. Tregs isolated in this manner are highly pure, can be expanded, and show effective *in vitro* and *in vivo* suppressive function (166). However, other data has shown CD49d^-^CD127^-^ isolated Tregs after *in vitro* expansion are less suppressive, exhibit lower levels of FOXP3 and TSDR demethylation than CD4^+^CD25^hi^CD127^-^ or CD4^+^CD25^hi^ICOS^+^ isolated cells (167). Despite this conflicting evidence, untouched Treg isolation protocols have been scaled up for the generation of GMP cell therapy products from large-scale leukapheresis samples, with the final product demonstrating <10% contamination with CD4 effector T-cells and <2% of all other cell types (168). Singapore General Hospital is currently preparing to conduct T-cell therapy trials using untouched Tregs in graft-versus-host disease following stem cell transplantation.

Another putative negative selection marker for Tregs is CD26, a widely distributed, 110-kDa, membrane-bound glycoprotein with intrinsic dipeptidyl peptidase IV activity. The ectonucleotidases, CD39 and CD73, in combination with adenosine deaminase (ADA), which degrades adenosine into inosine, together regulate pericellular adenosine concentrations. In humans, ADA is associated with the extracellular domain of CD26, a complex not seen in mice (169). The lack of anti-ADA antibodies suitable for flow cytometry necessitates CD26 analysis as a surrogate for ADA expression in T-cells. Despite a large amount of data supporting the costimulatory role of CD26 in T-cells (170–172), its exact function in Tregs has not been confirmed. The absence of CD26-ADA expression in Tregs was first described in 2010 (173). More comprehensive work in 2012 reported the presence of CD26-ADA on FOXP3-expressing activated CD4^+^ Teff cells, but not suppressive Tregs (174). In combination with markers like CD25, FOXP3 and CD127, CD26 may facilitate quantitative evaluation of Tregs or Treg isolation from samples containing activated Teff cells. Other studies have also described the use of CD26 as a negative selection marker for Tregs (175–178). However, further investigation is required to determine whether the CD26^-/low^ phenotype is a reliable method of isolating human Tregs and one that improves on current protocols.

CD6 and CD226 have not only facilitated the depletion of contaminants but also shed light on further subclassifications of tTregs. CD6 is a cell-surface glycoprotein predominantly expressed on T-cells (179, 180). It functions as a costimulatory molecule during TCR activation and plays a role in the responses of mature T-cells to both nominal antigens and autoantigens (181, 182). In 2014, Garcia Santana et al. identified CD4^+^CD25^hi^CD6^lo/-^ Tregs in man as FOXP3^+^ natural Tregs (nTregs), exhibiting *in vitro* suppressive activity on CD8^+^ T-cell proliferation. CD6 in combination with CD127 was postulated to serve as a tool to identify and isolate nTregs (183). Although CD6 has been reported as a negative marker for human Tregs in different studies, further investigation is needed to validate the utility of CD6 as a Treg exclusion marker (175, 184).

Although CD226, also known as DNAM-1, plays a critical role in immunoregulation, little was known about its cellular distribution in human Tregs until 2015, when Fuhrman et al. demonstrated that isolating CD226^-^ Tregs gave a highly pure and suppressive population. The CD226^+^ population is less pure and suppressive after expansion and demonstrated less demethylation in the TSDR locus, as well as significantly higher production of effector cytokines (185). Further work showed CD4^+^CD25^+^CD226^-^ cells, after 14 days of expansion, are more suppressive, produce more TGF-β1 and fewer effector cytokines like IFN-γ, TNF, and IL-17A than CD4^+^CD25^+^CD127^lo/-^ cells (186). Furthermore, CD4^+^ CD25^+^ CD226^-^ Tregs may not only be long-lived but also potentially localize more readily to secondary lymphoid organs. These data argue for CD226 as an important negative phenotypic marker of Tregs and the excluding CD226-expressing cells during Tregs sorting yields a population with increased purity, lineage stability, and suppressive capabilities, which may benefit Treg adoptive cell therapy. However, this approach should be taken with caution, as CD226 is also highly expressed by IL-10-secreting Tr1-like T-cells (187).

In addition to the aforementioned, CD154, also known as CD40L, can also be used to negatively identify activated Tregs. This member of the TNF superfamily is a recognized activation marker of CD4^+^ Teff cells (188, 189). The absence of CD154 expression in the presence of 4-1BB (4-1BB^+^CD154^-^) has emerged as an *ex vivo* and *in vitro* Treg activation signature, allowing for the identification and isolation of epigenetically stable, antigen-activated Tregs for rapid functional assessment (144, 145).

## 6 Key markers associated with the Treg function

Tregs exert their suppressive effects via a plethora of mechanisms, acting on various targets. These include modulating the cytokine microenvironment (85, 190, 191), metabolic disruption of target T-cells (8, 192), regulating the activation capacity of dendritic cells (193, 194), and direct cytolysis (195, 196). Building upon this, we next review previously discovered and newly identified markers of Tregs function.

Transforming growth factor-β (TGF-β), the most common isoform of which is TGF-β1, is a pleiotropic cytokine. First discovered in 1986, it was noted to induce anchorage-independent growth (197). TGF-β later became the primary focus of the immunosuppression and tolerance research field because of its potent inhibition of immune responses, impacting particularly upon T-cell proliferation and differentiation (198, 199). This was solidified when the connection between TGF-β and the suppressive function of murine Tregs was identified (77, 200), and the concept of TGF-β1 tethered to the cell surface of Tregs was introduced. Upon stimulation, CD4^+^CD25^+^ Tregs but not CD4^+^CD25^-^ conventional T-cells express high and persistent levels of TGF-β1 on the cell surface. However, the role of TGF-β in the immunoregulatory function of Tregs has sparked controversy, as previous studies had shown that neutralizing TGF-β using anti-TGF-β antibodies failed to reverse the suppressive function of Tregs *in vitro*. This was addressed in 2004 when it was reported that Latency associated protein (LAP) binds latent TGF-β1 in Tregs (201). Thus, TGF-β1-mediated immunosuppression occurs in a two-step process: first, TGF-β1 dissociates from LAP, then free TGF-β1 can interact with its receptor. This multistep process explains why antibody mediated TGF-β inhibition requires the high antibody concentrations necessary for TGF-β1 quenching. Crucially, expression of LAP on activated human FOXP3^+^ Tregs has since been shown to identify highly pure and functional Tregs from *ex vivo* expansion cultures (202, 203). For reviews on TGF-β in Treg biology, and the associated debates, see reviews by Tran and Moreau et al. (204, 205).

The cell surface molecule Glycoprotein A repetitions predominant (GARP), also known as LRRC32, was first reported in human Tregs by Wang et al. and has since been identified to be involved in TGF-β1 mediated Treg immunosuppression. TCR-activated Tregs exhibit significant upregulation of GARP on their cell surface, which has been linked to enhanced Treg suppressive capacity (206). Several groups have independently reported that GARP on Tregs binds latent TGF-β1, promoting the release of the activated TGF-β, leading to TGF-β mediated immunosuppression (207–209). Studies of Tregs in human disease have confirmed that TGF-β, GARP and LAP are vital to Treg function in inflammatory diseases (210) and cancers (211), highlighting the potential value of these receptors as immunotherapy targets.

Tregs can also suppress effector T-cells through adenosine binding to A2A receptors (212, 213). ATP is cleaved in tandem by two Treg-associated ectonucleotidases, CD39 and CD73, leading to adenosine production. Of the two ectonucleotidases, CD39, which hydrolyses ATP and ADP into AMP, is the rate-limiting enzyme (212). CD73, an ecto-5′-nucleotidase, exists in both soluble and membrane-bound forms and catalyzes the dephosphorylation of AMP into adenosine (214, 215). Adenosine then binds the A2A receptor on T-cells, leading to cAMP-mediated suppression of TCR signaling via PKA.

The role of adenosine in the function of human Tregs was discovered in 2010, introducing CD39 as a novel phenotypic and functional marker specific to human Tregs (173). In contrast to mice, which express CD39 constitutively on virtually all CD4^+^CD25^+^ T-cells, expression on human T-cells is restricted to a subset of FOXP3+ effector/memory-like Tregs (216, 217). Nearly all (>90%) CD4^+^CD25^hi^FOXP3^+^ adult human Tregs are CD39 positive, distinguishing human Tregs from other T-cell subsets. Due to its expression on the cell surface, CD39 has facilitated the successful and reliable isolation of functionally active human natural Tregs from peripheral blood (218). However, this approach should be taken with caution, as CD39 is not exclusive to Tregs and the CD4^+^CD39^+^ fraction of PBMCs also includes CD25^-^FOXP3^-^ T-cells (219, 220). Nevertheless, several studies have revealed that CD39^+^ Tregs demonstrate stronger stability and function under inflammatory conditions and superior suppressive capacity *in vitro* and *in vivo* (221–224), as well as an ability to suppress pathogenic Th17 responses (225).

CD73 was initially described as a characteristic surface marker of murine Tregs, however, it was only observed in a small proportion of human Tregs. Over 70% of CD4^+^CD25^hi^ Tregs express CD73 intracellularly, while the expression is limited to 20% of CD4+CD25- T-cells (173). These data indicate that CD73 is readily internalized from the surface of human lymphocytes (226, 227) and is predominantly cytosolic in human Tregs. However, expanded human CD4^+^CD25^hi^CD127^lo^ cells show a higher surface expression of CD73 (~35%) as compared to unstimulated memory Tregs (CD4^+^CD25^hi^CD127^lo^CD45RA^-^) (~5%) (228). These expanded Tregs are highly suppressive, attributable to the surface expression of CD73 along with CD39. Additionally, CD73 on murine Tregs has been shown to suppress proliferation and cytokine secretion by T helper 1 (Th1) and Th2 cells (229). Taken together, these results strongly indicate the physiological importance of CD39 and CD73 expression in Tregs (228, 230).

## 7 Emerging markers of Tregs function

**Figure 2:**
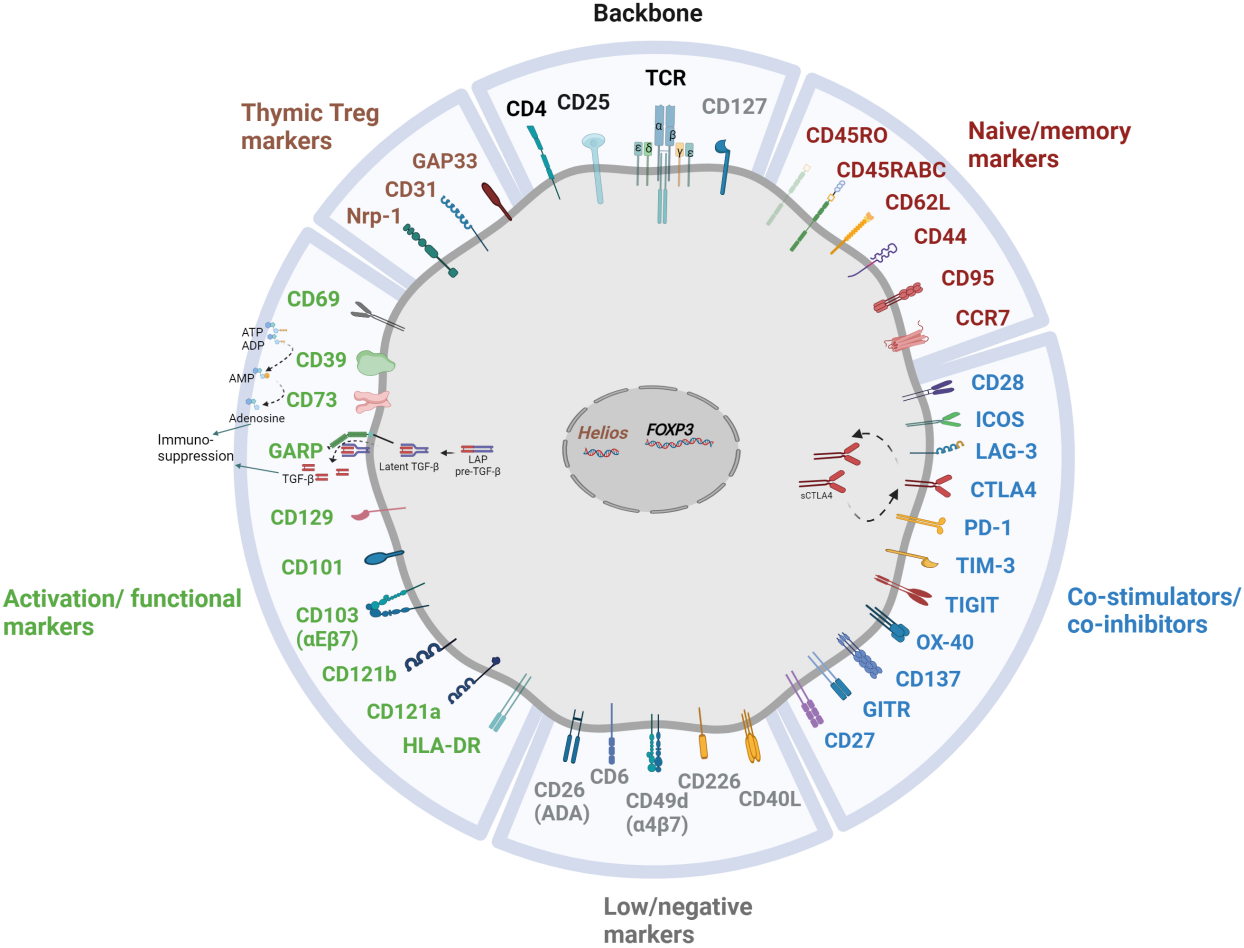
Multitude of markers illuminating the heterogeneity of Treg subpopulation.

As previously discussed, a multitude of Treg markers are associated with their origin, maturation, stability, and functional characteristics, as concisely delineated in **Figure 2**. In recent years, several emerging markers have garnered attention for their potential contributions to Treg-mediated immunosuppression. Although the precise roles and mechanisms of action remain a subject of ongoing investigation, preliminary research suggests their potential significance in modulating immune responses.

CD121a and CD121b are two unique cell-surface antigens on human Tregs that are transiently expressed upon TCR-mediated Treg activation, distinguishing functional Tregs from activated FOXP3^-^ and FOXP3^+^ non-Tregs, alongside LAP (202). These two highly specific surface markers were recommended for high yield, high purity Treg isolation, promising a rapid advancement in the therapeutic application and functional analysis of Tregs in human disease. However, the popularity of these two markers has waned due to a lack of subsequent studies that could verify these properties.

CD69 and HLA-DR are widely recognized as activation-induced cell surface markers in both conventional T-cells and Tregs, as well as markers of Treg differentiation and immunosuppression. CD69, upon binding with its ligand the S100A8/S100A9 complex, regulates Treg differentiation (231). Further reports indicate CD69 expression may be essential for tTreg development (232). Moreover, FOXP3^+^CD69^+^ Tregs express higher surface levels of suppression-associated markers and display enhanced suppressive activity as compared to FOXP3^+^CD69^-^ Tregs (233, 234). CD69 also enhances the immunosuppressive function of Tregs by prompting IL-10 production (235). However, due to the lack of knowledge regarding the functional roles of CD69 in human Tregs, no definitive conclusions can be drawn at this time.

Approximately one-third of adult human peripheral blood CD4+ effector Tregs express HLA-DR, identifying a distinct and highly functional subset of terminally differentiated Tregs (236). HLA-DR^+^ Tregs exhibit higher levels of FOXP3 and employ contact-dependent immunosuppression, indicating superior suppressive capabilities (237, 238). However, despite HLA-DR+ Tregs being regarded as superior in functionality (239–241), there is insufficient data demonstrating HLA-DR as a marker of Treg function.

Two other cell surface receptors, CD101 and CD129, have been linked with Treg suppressive activity (242). Earlier mouse studies revealed that CD101 surface expression is strongly correlated with suppressive activity in CD4^+^CD25^+^ Tregs, both *in vitro* and *in vivo* (243). However, studies in human Tregs have not replicated this observation (244). Similarly, IL-9 generated by activated T-cells promotes the proliferation of Th clones and enhances the suppressive function of CD129 (the IL-9 receptor) expressing Tregs (245, 246). However, these two markers have lost popularity given the lack of evidence for them in man.

CD103, also known as integrin αEβ7 (ITGAE), is expressed on approximately 10-30% of mouse pTregs. Whilst not indicative of activation status, its levels can be upregulated in inflamed tissues (247–249). CD103^+^ Tregs have been found to exhibit slightly higher suppressive capacity and express higher levels of IL-10, contributing to their anti-inflammatory activity (132). Other findings in mice include the ability of CD103^+^ Tregs to restrain CD8^+^ T-cell activation (250). Human CD25^+^FOXP3^+^ Tregs, however, show limited expression of CD103 in various tissues, including blood (<5%) (251–254). Nevertheless, human CD4 T-cells can be induced to express CD103 through various stimuli *in vitro* (255, 256) and the percentage of CD103^+^ cells among CD4^+^ CD25^+^ T-cells is significantly higher than CD4^+^CD25^−^ T-cells in patients with multiple sclerosis (251). Further investigation is needed to determine whether CD103 is upregulated on human Tregs in other inflammatory conditions.

Recent studies have identified the potential expression of CD70 (157), CD80, and CD86 (257) in Tregs, opening new possibilities for identifying novel Tregs subsets and markers of their potential therapeutic efficacy.

The summary of all the markers discussed above with their alternative designations, and their patterns of expression in human Tregs is presented in **Table 1**.

**Table 1:** Markers of human functional FOXP3^+^ Tregs.

Markers	Alternative name(s)	Marker location	Expression on resting Treg	Expression level upon activation
CD25	IL-2Rα	Surface	CD25^high^	Very high
FOXP3	N/A	Intranuclear	FOXP3^high^	Very high
CD127	IL-7Rα	Surface	CD127^low/-^	Low/-
CD45RA	N/A	Surface	Naïve Treg are predominantly CD45RA^+^	Low/-
CD45RO	N/A	Surface	Memory Treg are predominantly CD45RO^+^	High
CD62L	L-selectin	Surface	Treg with secondary lymph node homing capacity are predominantly CD62L^+^	Retained
CCR7	CD197	Surface	Most of the Treg with secondary lymph node homing capacity are CCR7^+^	Retained
CD95	Fas	Surface	Most of the naïve Treg are CD95^low/-^	High
CD44	N/A	Surface	Most of the Treg are CD44^low/-^	High
CD31	PECAM-1	Surface	Recent thymic emigrant Treg are CD31^+^	Low
Helios	IKZF2	Intranuclear	Thymus derived stable Treg are Helios^+^	Stable over time
GPA33	A33	Surface	Pure and stable naïve Treg population, that are also derived from thymus, are GPA33^+^	low
CTLA-4	CD152	Surface/Intracellular	Treg express CTLA-4 constitutively at low level	High
PD-1	CD279	Surface/Intracellular	Most of the PD-1 expressing Treg retain its expression in the intracellular compartments	High (surface)
ICOS	CD278	Surface	About 20% of the peripheral Treg are ICOS^+^	High
TIGIT	VSTM3	Surface	Few Treg with activated phenotype are TIGIT^+^	High
LAG-3	CD223	Surface	Most of the resting Treg are LAG3^low/-^	High
TIM-3	CD366 HAVCR2	Surface	Only 2-5% of Treg in periphery are TIM-3^+^	High
GITR	TNFRSF18 CD357	Surface	Few Treg with activated phenotype are GITR^+^	High
OX-40	CD134	Surface	Few Treg with activated phenotype are OX-40^+^	High
4-1BB	CD137	Surface	Few Treg with activated phenotype are 4-1BB^+^	High
CD27	N/A	Surface	Treg with high suppressive capacity are CD27^+^	Mostly stable over time
CD49d	α4 integrin VLA-4 α	Surface	70% of the immunosuppressive FOXP3+ Treg are CD49d^low/-^	High
CD26	ADA binding protein	Surface	Most of the Treg are CD26^low/-^	High
CD6	–	Surface	Most of the Treg are CD6^low/-^	High
CD226	DNAM-1	Surface	Most of the Treg are CD226^low/-^	High
TGF-b	TGF-b1, LAP	Surface/Intracellular	Most of the resting Treg are TGF-b^low/-^	High
LAP	TGF-b1	Surface/Intracellular	Most of the resting Treg are LAP^low/-^	High
GARP	LRRC32	Surface	Most of the resting Treg are GARP^low/-^	High
CD39	N/A	Surface	Only effector/memory-like Treg are CD39^+^	High
CD73	N/A	Surface/Intracellular	Most of the CD73 expressing Treg retain in the intracellular compartments	High (surface)
CD121a/b	IL-1R I/II	Surface	Most of the resting Treg are CD121a/b^low/-^	High (transient)
CD69	N/A	Surface	Most of the resting Treg are CD69^low/-^	High
HLA-DR	N/A	Surface	Few Treg with activated phenotype are HLA-DR^+^	High
CD101	BB27	Surface	Less studied in humans	N/A
CD129	IL-9R	Surface	Less studied in humans	N/A
CD103	Integrin αEβ7	Surface	<5% Treg in various tissues, including blood are CD103^+^	N/A
Neuropilin-1	CD304	Surface	Thymic derived mouse Treg are Neuropilin-1^+^ (Human studies are controversial)	N/A
CD70	CD27L	Surface	Only 5-6% Tregs are CD70^+^	High
CD80/CD86	B7 1/2	Surface	20% Treg are CD80^+^ and <1% Treg are CD86^+^	High

References are indicated in the text.

The table above presents an overview of human Treg markers that delineate different subsets of Treg cells, along with their respective properties, as detailed in the review.

## 8 The functional role of chemokines and chemokine receptor interactions in Treg migration

**Figure 3:**
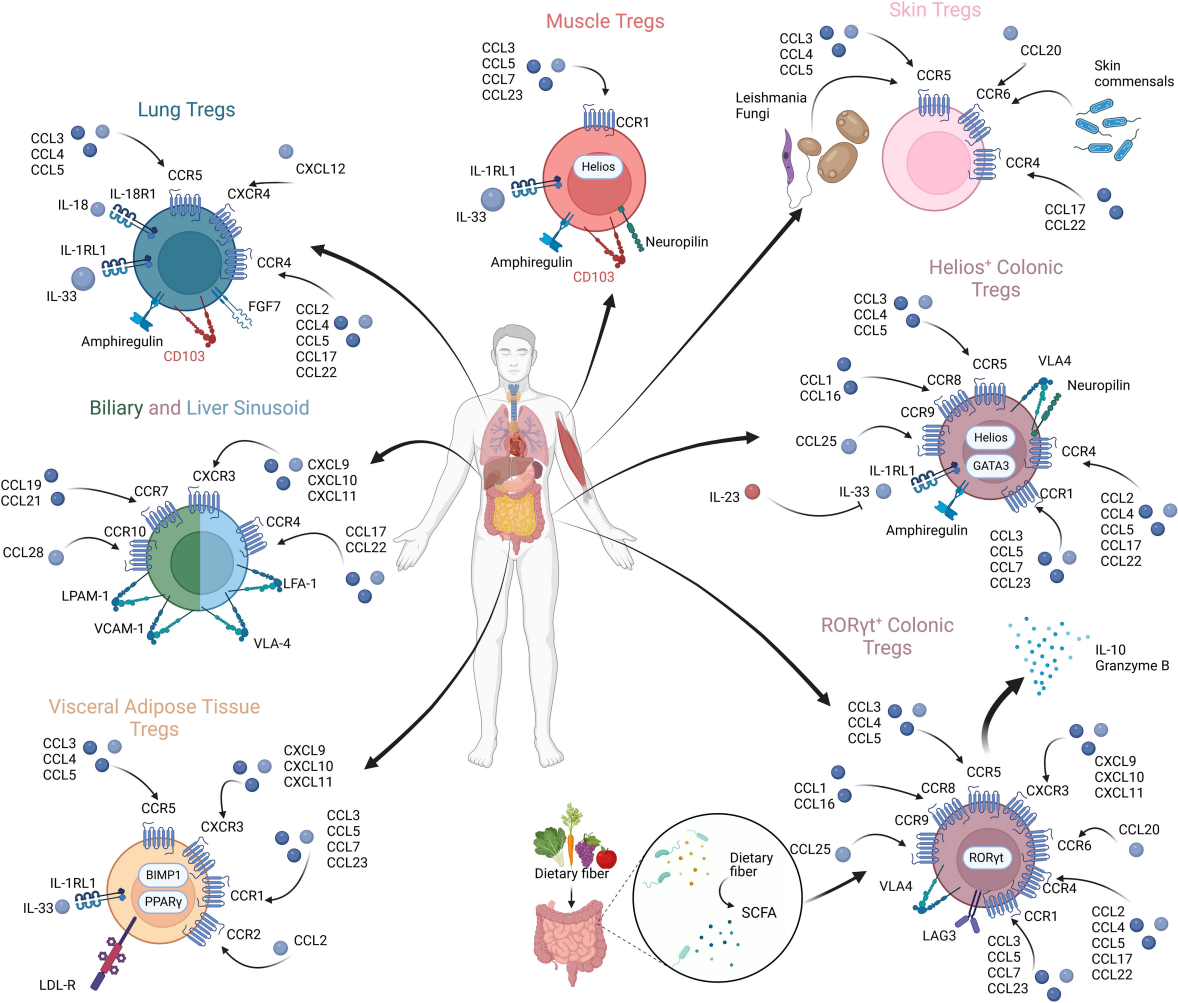
Tregs in diverse anatomical compartments exhibiting differential chemokine expressions and chemokine receptor profiles.

Chemokines are a group of small heparin-binding proteins that direct the movement of circulating immune cells, influencing their migration within inflamed tissues (258, 259). When initially identified, chemokines were described to be associated with inflammatory diseases (260–262). Later, their involvement in the migration of immune cells was identified (263), followed by production by immune cells themselves (264, 265). A plethora of studies have shown how perturbations in the distribution of Tregs can lead to organ-specific inflammatory diseases (258, 266–275). Chemokines govern the trafficking and homing of Tregs, and understanding how Tregs reach their site of action sets the ground for targeting the pathogenic Treg distribution in the context of cancer and may facilitate tissue-specific targeting in the context of Treg therapy. Variations in chemokine expressions across different human organs is summarized in **Figure 3**.

Several integrins have been identified to be crucial to Treg function and localization. The αvβ8 integrin is key to activating latent TGF-β and Tregs lacking αvβ8 are unable to suppress T-cell-mediated inflammation *in vivo* (276, 277). The α4β7 integrin (also known as LPAM-1 and whose classical ligand is MAD-CAM1) is recognized to play a crucial role in the migration of Tregs to the intestine and associated lymphoid tissues. Loss of the β7 has been shown to lead to disrupted migration, with subsequent colitis observed (266, 278, 279). The expression of the α4β7 integrin and CCR9 enables Tregs to home to the gut’s lamina propria, allowing for tolerance to food antigens, crucial to maintaining gut health (266, 267).

### 8.1 Tregs in lymphoid tissue

Tregs play a major role in constraining immune response in secondary lymphoid organs, with CCR7 being one of the first chemokine receptors identified on Tregs (30, 280). CD62L^hi^CCR7^+^ naïve Tregs favor migration into secondary lymphoid organs where professional antigen presentation allows for antigen-dependent stimulation, modifying the pattern of receptor expression to enable tissue homing (30, 30, 33, 281, 282). Lymph nodes draining these organs act with local dendritic cells to induce Treg activation and action, while CCR7 is downregulated, promoting accumulation in inflamed tissues (38, 283–285). Notably, the loss of CCR7 results in impaired *in vivo* Treg suppressive activity, while their *in vitro* activity remains intact. This highlights the significance of CCR7 to Treg function through facilitating localization (33, 38, 286, 287).

Native Th1 responses are dependent on CXCR3-mediated signals. Tregs inhibit stable contacts between CD4^+^ T-cells and dendritic cells and can influence the expression of CXCR3 ligands in draining lymph nodes. Early data demonstrated that Treg localization in the lymph node, when suppressing CD4^+^ T-cell responses, was via localization with dendritic cells, rather than the CD4^+^ T-cells, suggesting they act by inhibiting the dendritic cell-CD4^+^ T-cell interaction (193, 194). Further data has suggested, Tregs can also prevent the expression of CXCR3 on effector T-cells, inhibiting their migration and trafficking (288, 289). Tregs can also upregulate T-bet and express CXCR3 in response to INF-γ. These CXCR3 expressing Tregs not only resemble Th1 subsets of effector T-cells, expressing T-bet in addition to FOXP3 but also demonstrate an ability to produce INF-γ and IL-10. Crucially, they are essential to the suppression of Th1-mediated inflammation, and imaging data suggests their function is mediated by colocalization with effector Th1 CD4^+^ and CD8^+^ T-cells in secondary lymphoid organs (36, 290–292).

CCL3 and CCL4 interactions with CCR5 are recognized to be important to CD4^+^ and CD8^+^ T-cell interactions in the lymph node for generating memory CD8^+^ T-cells (293). The production of CCL3 and CCL4 by Tregs has been observed, attracting CD4^+^ and CD8^+^ T-cells via CCR5 expressed on these cells; bringing them into proximity and allowing Tregs to exert their suppressive function. Tregs deficient in CCL3 and CCL4 fail to prevent the progression of experimental autoimmune encephalomyelitis or islet allograft rejection in murine models and Tregs from individuals with type 1 diabetes demonstrate an impaired ability to produce CCL3 and CCL4 (268). Additionally, Tregs can interfere with the priming of CD8^+^ T-cells by modulating the expression of the CCR5 ligands CCL3, CCL4 and CCL5. The deletion of Tregs leads to the stabilization of interactions between dendritic cells and low-avidity T-cells, compromising the responses of high-avidity memory T-cells and memory responses (289, 294).

CXCR5 is key to the function of Tregs in modulating B cell and humoral responses. Chung et al. were able to show Tregs expressing CXCR5 and Bcl-6 localize to germinal centers, with CXCR5 expression being Bcl-6 dependent (295). These follicular Tregs, are absent in the thymus. Their depletion leads to exaggerated germinal center reactions, including germinal center B cells, affinity maturation of antibodies and the differentiation of plasma cells (289, 295). These results were confirmed by work by Linterman et al., which showed follicular Tregs regulate B cell responses, controlling germinal center responses (289, 296–299). Follicular Tregs are hypothesized to exert their effect via various mechanisms, including the production of IL-10, and TGF-β (300–302). Intriguingly, CXCR5 is not necessary for the localization of follicular Tregs, but the transcription factor Bcl-6 is necessary for function and localization (36, 297, 298, 303, 304).

Finally, a CCR6^+^ Treg subset has recently been demonstrated to be significant to thymic Treg development and function. These IL-1R2-positive Tregs express CCR6, but not CCR7, which suggests they recirculate from the periphery with an activated phenotype. These Tregs can quench IL-1, indicating they can maintain tTreg development even in inflammatory conditions (305).

### 8.2 Tregs in various tissues

Tregs in various tissues demonstrate specificity to site and function. Here we describe selected examples of organ-specific Tregs and the chemokine receptor profiles that facilitate and define their tissue tropism, allowing immunosuppression within their unique environments (285, 306).

Visceral Adipose Tissue: Tregs in visceral adipose tissue (VAT) show enrichment in CCR1, CCR2, CCR5 and CXCR3, as well as the IL-33 receptor, ST2 (also known as IL-1RL1) (307–309). Tregs in visceral adipose tissue migrate under the influence of the IL-33/ST2 axis, with the majority of IL-33 arising from mesenchymal stromal cells (310, 311). Hydroxyprostaglandin dehydrogenase expressed by VAT Tregs exerts an inhibitory effect on conventional T-cells and inflammation (312). Most interestingly, however, is the limited range and repertoire in Treg TCRs present on VAT Tregs, suggesting they respond to specific antigens found in the tissue of residence (275, 309, 313). This may relate to their function in regulating inflammation within VAT, and thus having a restricted repertoire of antigens which they may need to respond to, as it appears VAT Tregs regulate the inflammatory state and promote insulin sensitivity (314). Knockout of this subset of Tregs leads to impaired function in the insulin receptor and chronic inflammation (314).

Skin Tregs: A significant subset of peripheral blood Tregs appear to express CCR4, but Tregs in certain tissues seem to upregulate the expression of this receptor, with its presence noted in skin, liver, lung and intestinal Tregs (315–317). The loss of CCR4 on murine Tregs impairs Treg accumulation in skin and lung tissues, with complete loss of CCR4 in the Treg cell compartment leading to severe dermatitis, pneumonitis and lymphadenopathy (316).

CCR5 has been shown to be key to the function of many tissue resident Tregs, as well as potentially having a role in persistent infections. In a *Leishmania major* model, CCR5 was essential to the homing of Tregs to *Leishmania* infected dermal sites, promoting infection and parasite survival (318). Similar results have been observed in chronic fungal granulomata, with chronic inflammation mediated by CCR5 expressing Tregs, as pathogens generate CCR5 ligands, leading to Treg recruitment and dampened immune responses (270).

In neonates, CCR6 is crucial to Treg tropism to the skin. Interactions in early life between commensals and CCR6 expressing Tregs induce Treg accumulation at this site, facilitating tolerance to normal skin commensals, mediated by CCL20 and CCR6 interactions (319, 320).

Skeletal muscle and regulatory T-cells: Tregs in muscle are characterized in part by the presence of CCR1 on the surface, as well as ST2 and the key growth factor Amphiregulin (271). These Tregs seem to demonstrate a key role in promoting muscle repair and regeneration (271, 272, 321–323). Tregs in muscle are predominantly recruited from the circulation via the IL-33/ST2 axis, as is seen in VAT Tregs (321). Once in the muscle, CD103 plays a key role in adherence and retention of these Tregs (324). Interestingly, muscle Tregs seem to show a high level of Helios and Neuropilin, suggesting a thymic origin, however, data clearly shows their recruitment from the circulation. Similar to VAT Tregs, muscle Tregs demonstrate a limited repertoire of TCRs, suggesting specific and limited targets (271). They are recognized to be involved in the repair and regeneration of muscle tissue, having been identified in the inflammatory infiltrate of injured muscle, both in acute and chronic muscle injury, expressing IL-10 where observed (271, 272). Indeed, muscle Tregs were shown to decline with age which likely contributes to defects in muscle regeneration seen in aging (325). Amphiregulin seems to play a crucial role in the regeneration of muscle cells, acting directly on satellite cells, promoting myocyte differentiation *in vitro* and enhancing muscle repair *in vivo* (271, 272, 275, 308, 326).

Lung Tregs: Tregs found in the lung, as discussed above, predominantly demonstrate upregulation in CCR4 (327). Similar to muscle Tregs, Tregs in the lung also appear to have a high expression of amphiregulin. Distinct to these Tregs however is the expression of IL18R1, ST2, CCR5, CXCR4, and KGF with downregulation of CXCR5 upon activation (307, 328). Tregs are recruited to the lung via TGFβ expression by a resident IFNAR1^hi^ TNFR2^+^ conventional dendritic cell 2 (iR2D2) population (329). Tregs play a crucial role in the resolution of lung injury, modulating immune responses and enhancing alveolar epithelial proliferation and tissue repair through the expression of amphiregulin and keratinocyte growth factor (KFG) (330). Inflammatory responses are also dampened by the upregulation of Mmp12, inhibiting neutrophil recruitment. Sik1, meanwhile, has been shown to be downregulated on activation of lung Tregs, leading to an increase in the expression of CD103 (αEβ7) in response, facilitating Treg adhesion and tissue retention (328). Furthermore, Tregs have been highlighted to play a role in asthma. The Notch4 pathway has been found to be upregulated in Tregs in asthmatics. Defective Hippo signaling gives rise to FOXP3 CN2 methylation in Tregs, impairing Treg stability and thus their ability to regulate inflammation in this environment. Thus, the rescue of Tregs in this environment and restabilizing them presents a future therapeutic direction in asthma (273).

Tregs in the gut: The gut provides a dynamic environment, containing a complex interplay of food antigens and resident microbial antigens. As the principal digestive and absorptive site in mammals, an immense range and variety of peptides are present. There is a complex interplay in the gut of various resident and migrating immune cells, from both innate and adaptive systems. Regulating these interactions is essential, and Tregs play a crucial role in orchestrating responses (331). The ablation of colonic Tregs has been demonstrated to lead to aberrant Th1 and Th17 mucosal responses, which are rescued with adoptive transfer (274, 275, 332). Chemokine receptors in gut resident Tregs include CCR1, CCR4, CCR5, CCR8, CCR9 and CXCR3, in addition to the integrin α4β7 (discussed above) (306, 307, 333). This subset of Tregs play a key role in the regulation of complex immune interactions and have been found to perhaps represent two distinct populations, one derived from the thymus and another from peripheral Tregs.

Single-cell transcriptomic data has since shown the colon has populations of tTregs, reflecting those seen in non-lymphoid tissues (expressing GATA-3, neuropillin-1, amphiregulin, ST2 and Helios), and suppressive pTregs (expressing IL-10, Granzyme B, LAG3 and CXCR3) defined by the expression of the transcriptional factor RORγt (306). Interestingly, the microbiome seems to play a crucial role in the recruitment of the latter, being totally absent in germ free mice, and bile acid metabolites impact their function and proliferation (334–337). The two first reports of RORγt Tregs were published together in 2015 (335, 336). Crucially, the data on the function of these cells was divergent, with one group finding that they inhibit Th1/17 responses, while the other demonstrated they respond to Th2 cells. Others have shown that RORγt expression in Tregs may represent a step in the process towards a Th17 Treg phenotype, expressing CCR6 and sharing phenotypic features usually observed in Th17 T-cells (97, 338).

IL-33 has been noted to be present in high quantities in human inflammatory bowel disease, as well as colonic inflammatory lesions. This binds ST2 on Tregs, stimulating responses by enhancing TGF- β1-mediated differentiation and providing signals for the accumulation and maintenance of Tregs in inflamed tissues. Conversely, IL-23, inhibits IL-33 responses. These data suggest that ST2-mediated responses and the balance between IL-33 and IL-23 may contribute to the control of intestinal immune responses (339). Additionally, Blimp-1 has been shown to control the differentiation and function of Tregs, promoting IL-10 generation and ICOS expression in Tregs and suppressing colitis via an IRF4-dependent pathway (340, 341).

Treg/Th17 imbalances are well established to be at play in the pathogenesis of ulcerative colitis. CCR6 Tregs have been shown to be enriched in ulcerative colitis patients and correlated with disease activity. CCR6^+^ Tregs also demonstrated a higher propensity to secrete IL-17A, suggesting that they may play a part in the ongoing colitis (269). RORγt expressing Tregs have been noted to express CCR6 and this may be a marker of Th17 biased Tregs (338).

In addition to the above, a novel chemokine receptor, GPR15, an orphan GPCR which binds to its own ligand (GPR15L) has been shown to play a role in the trafficking of Tregs to barrier tissues, including skin and colon, where it is involved in the suppression of colitis and graft rejection. GPR15 has been shown to have its expression modulated by both gut microbiota and TGF-β, with knockout of this receptor giving rise to colitis, rescued by transfer of Tregs with intact GPR15, suggesting GPR15 has a crucial role to play in mucosal health (36, 342, 343).

Liver Tregs: The liver, is a key organ to gut health and homeostasis. With a dual blood supply, and 75% of this from the portal system, it attracts a high burden of various food and microbial antigens and consequently, a tolerogenic environment has to be maintained. However, appropriate responses must be mounted to remove pathological antigens and particulates, preventing them from entering the systemic circulation (344).

CXCR3 is critical to Treg migration into the liver and liver sinusoids. This chemokine receptor allows for the colocalization of Tregs within the liver, with CCR4 cognate ligands (CCL-17 and -22) allowing for colocalization with infiltrating dendritic cells within inflammatory infiltrates in liver diseases (344, 345). Diseased livers demonstrate elevated expression of CXCR3 on Tregs (258). This adhesion is mediated by CXCL-9, -10 and -11 expression. *In vitro* models have demonstrated the upregulation of this chemokine under stress (346). Contacts made by lymphocytes and sinusoidal epithelial cells activates integrins, including LFA-1 and VLA-4 (also known as α4β1) on lymphocyte. These integrins engage adhesion molecules, including ICAM, VCAM and VAP-1 on epithelial cells, facilitating Treg transmigration into the hepatic parenchyma (347). This process enables Tregs to access the hepatic sinusoidal environment and may be utilized for clinical applications, as described below (258).

Additionally, CCL28 production in the biliary epithelium recruits CCR10 expressing Tregs. CCL28 is secreted by primary human cholangiocytes in response to LPS, IL-1 and bile acids. The CCL28 - CCR10^+^ interaction induces adhesion molecule expression, including VCAM-1, and α4β7. Furthermore, CCR10^+^ Tregs exhibit low expression of CCR7 (indicating a memory phenotype) and high levels of CXCR3 (348).

In inflamed bile ducts, CCL20 is secreted by biliary epithelial cells. In a Th17 inflammatory environment, replicated *in vitro* by a cocktail of IL1β, TNFα, IFNγ and IL-17, CCL20 is secreted by human biliary epithelium and leads to recruitment of CCR6 chemokine receptor expressing effector T-cells to sites of biliary inflammation (97, 349, 350). Additionally, CCR6 is a common Treg marker, associated with Th17 skewing. However, in the liver, CCR6 Tregs are liable to phenotypic instability and conversion to a Th17 inflammatory phenotype. This instability challenges their viablility as a candidate for clinical application or cell therapeutic (258). Additionally, it has been noted that CCR6 expressing Tregs in the colon can mount Th17 responses, regulated by IL-10 production in CCR6 expressing Tregs (97, 351, 352).

By demonstrating tissue selectivity and specificity through chemokine receptors, Treg cell products can be designed for organ-specific cell therapy. Liver diseases such as primary sclerosing cholangitis, primary biliary cirrhosis, autoimmune hepatitis and liver transplant rejection all demonstrate a significant contribution from T-cell immune responses to autoantigens expressed in either the biliary epithelium or hepatocytes (353–365). The proof-of-concept AUTUMN study (Autologous regUlatory T-cells infUsion and tracking in autoiMmuNe hepatitis) employed GMP-manufactured Tregs and found infused Tregs favoured migration to the liver (366).

Tregs in the Bone Marrow: Tregs expressing CXCR4 have been shown to collect in the bone marrow, with loss of CXCR4 impairing accumulation in the bone marrow. The depletion of CXCR4 Tregs has also been shown to lead to an increase in B1 mature B cells, exclusively within the bone marrow, with no observed changes in plasma cells or hematopoietic stem cells and no signs of immune activation elsewhere. However, the loss of these CXCR4 Tregs does lead to an increase in total serum IgM levels and a highly specific increase in IgM autoantibodies (367).

### 8.3 Chemokine receptors in cancer

Tregs are enriched in the tumour microenvironment, and their enrichment has been correlated with worse outcomes (368). Tregs in the tumour microenvironment supress host immune responses, subverting immune destruction and thus supporting tumor growth (369). Consequently, Tregs have become a focus of cancer immunotherapy (370). However, specifically targeting cancer facilitating Tregs and Tregs in the tumour microenvironment remains a barrier to effective cancer immunotherapy with limited offsite side effects (371, 372).

CCR4 is predominantly expressed in tumor resident Tregs, enabling tumor immune evasion, and is thus regarded as a potential candidate target for cancer immunotherapy (71, 373, 374). CCR8’s more recent identification as a chemokine receptor specifically upregulated in cancer Tregs and it’s correlation with poor outcomes in patients has garnered significant interest (36, 375, 376). This cytokine receptor has been shown to be upregulated in colorectal, non-small cell lung, breast, and other cancers. Additionally, some data has shown CCR8 to be upregulated upon Treg activation, with autocrine production of CCR8’s ligand CCL1 (377). Data on targeting CCR8 in cancer thus far, however, has been conflicting, but some preclinical models have shown promise (378–381).

Nevertheless, both CCR4 and CCR8 appear to be expressed under the control of GATA3 and IRF4, both of which are canonical Th2 transcription factors (36). The expression of these transcription factors on activated Tregs hints at a Th2 skewed Treg population and suggests that CCR4 and CCR8 may be chemokines specific to Th2 Treg function (382–384). GATA-3, meanwhile, has been shown to be essential to Treg function, binding to the CNS2 in Foxp3 promoting Foxp3; with loss of GATA-3 leading to a 50% reduction in FOXP3 mRNA transcripts compared to baseline, with the same seen in other Tregs signature genes (CD25, CTLA-4, GITR). Furthermore, GATA-3 deficient Foxp3 Tregs acquire Th17 cytokine expression profiles (383, 385).

CCR6 has been shown to be a target of certain cancer-derived factors, promoting Treg migration. Eomesodermin has been shown to be generated by oesophageal adenocarcinoma, and this has been shown to drive CCL20 secretion, which binds to CCR6 on Tregs driving Treg chemotaxis and residency intratumorally, promoting cancer growth (386).

### 8.4 Chemokine receptors in graft-versus-host disease

GVHD is a complication affecting every second stem cell transplanted patient. Both acute and chronic GvHD are characterised by a systemic inflammation of recipient tissue caused by donor cells in the graft. Severe treatment refractory GvHD can often be lethal. Polyclonal Tregs have successfully been used in named patient programs and clinical trials by us (Trzonkowski; Fuchs(Theil)) and others, both to prevent and to treat GVHD (387–393).

In a murine model of GVHD, the knock-out of CCR5 impaired the migration of donor Tregs to GVHD target organs, leading to lethal outcomes (394). Additionally, CCR8 in GVHD has been shown to promote Treg maintenance, by allowing tolerogenic interactions with donor CD11c+ antigen presenters. In a murine model of islet transplantation, CCR5 along with CCR2, CCR4 and P- and E-selectins were essential to the movement of Tregs from blood into pancreatic islet allografts. Subsequent migration to lymph nodes was found to be CCR2, CCR4 and CCR7 dependent, from which they could inhibit effector T-cell responses in both the allograft and lymph node (395).

## 9 Genetic engineering to enhance Treg function

Deep understanding of Treg biology, mechanisms of immunosuppression, as well as identification of chemokine receptor patterns responsible for tissue tropism are cornerstones for the generation of genetically modified cells with improved function and enhanced infiltration of the target tissue. Polyclonal autologous Tregs have demonstrated efficacy in several trials, but accumulating evidence from preclinical models demonstrate increased potency of antigen-targeted approaches (1) (388, 390, 396–399). Recent success and FDA/EMA approval of chimeric antigen receptor (CAR)-T-cells in hematological cancers has caused a paradigm shift (400), with increased interest in CAR-Tregs. For example the technology enables generation of Tregs that will precisely target HLA mismatches in transplantation or antigens within inflamed tissues, facilitating direct local suppressive effects. CAR constructs in general are built from: 1) an extracellular ligand binding domain which consists of the antigen-specific variable fragment of heavy and light antibody chains (single-chain variable fragment; scFv), 2) hinge region (providing scFv flexibility), 3) transmemberane domain (that anchors the receptor in plasmalemma) and finally 3) an intracellular costimulatory domain derived from T-cells (401, 402). Until now five generations of CARs have been developed and tested with the main difference in the intracellular domain. The first-generation CARs comprised CD3ζ, a part of the T-cell receptor-CD3 complex, while second-generation CARs combine CD3ζ with costimulatory molecules such as CD28, CD137, CD27 or CD134 delivering a second signal and were the most extensively studied approach. The third-generation CARs comprise of two costimulatory molecules (403–405), fourth generation CARs co-express cytokine or chemokine genes, While the fifth generation receptors contain intracellular domain of a cytokine receptor (e.g., IL2RB chain) that interacts with STAT3 (406–409). In case of genetic Treg modifications, mostly 2^nd^ generation CARs were tested. The Levings´group conducted a comprehensive study exploring the Treg immunosuppressive capacity and stability after incorporation of various costimulatory domains into the CAR (including CD28, ICOS, CTLA4, PD1, GITR, OX40, CD137, and TNFR2) (410). Interestingy, in contrast to anti-tumor conventional CAR-T cells, Tregs with CD28-encoding CAR exhibited superior in vitro and in vivo performance in terms of proliferation, suppression, and delaying GvHD symptoms. In addition, both CD137- and TNFR2-CARs were found to negatively affect Treg function and stability, leading to FOXP3 locus methylation, decreased Helios expression, and reduced suppressive function in vitro and in vivo. Interesting the group published very recently, that Tregs expressing CARs encoding CD28, ICOS, PD1, and GITR, but not 4-1BB or OX40, all extended skin allograft survival in an immunocompetent transplant model (411). With other studies (412, 413) consistently reporting adverse consequences of using CD137 in Tregs, one may conclude that this co-stimulatory domain is suboptimal for CAR-Tregs. However, in the case of flexible modular chimeric antigen receptor technology called universal CAR (UniCAR-Tregs), both CD137 and CD28 costimulation induced robust suppressive capabilities. Nevertheless, due to higher background activation of Treg in case of CD28, UniCARs featuring a CD137-CD3ζ signaling domain were discussed as the preferred constructs for the clinical application of redirected Tregs (414). An intriguing avenue for exploration involves combining CD28 and CD137 domains to potentially optimize CAR-Treg suppression, as reviewed by Zhang, Qunfang et al. (415).

Noteworthy, IL-10 secretion was successfully induced into 4^th^ generation CAR-Tregs, enhancing suppressive function (416). However, no reports on fifth- generation CAR-Tregs have been available yet and their specific effects on CAR-Tregs needs further investigation. The state of knowledge on CAR-Treg generation is summarized in a review by M. Levings, 2020 (417). Further innovative adaptations of CAR designs have emerged with the potential to enhance the durability, stability, proliferative capacity, and function of CAR-Tregs. Additionally, novel CAR-Tregs specific for E.coli-derived flagellin have been designed and introduced into humanized murine models. These FliC-CAR-Tregs were directed to the colon (which confirmed improved homing abilities) and were characterized by higher expression of PD-1, highlighting higher immunosuppressive capacity and potential for application in inflammatory bowel diseases (418). Adoptive Treg therapy is limited by the plasticity of Tregs, potentially transforming into conventional T-cells. To overcome this challenge, additional genetic engineering has been suggested to enhance Treg stability and robustness. Examples include engineered epigenetic and post-transcriptional changes in FOXP3 and metabolic stabilization by enhancing CD39 and CD73 expression. Elevating IL-10, TGFβ, IL-34, IL-35, and FGL-2 can boost Treg function, while suicide genes help manage Treg adverse effects. This idea of the next generation of Super-Tregs with increased function, stability, redirected specificity and survival is summarized by Amini et al. (419).

Engineered antigen-specificity has demonstrated improved responses in mouse models, emphasizing utility (420, 421). In mouse models, researchers demonstrated that lentiviral transduction did not alter the Treg phenotype and HLA-A2 CAR-Tregs were effective in suppression. CAR-Tregs produced small amounts of IFN-ɣ, but more importantly, secreted the highest amounts of IL-10. Finally, transduced cells migrated more rapidly through HLA-A2+ HUVECs and contributed to the survival of human skin grafts (416).

Currently, CAR-Tregs remain under investigation (422), and two clinical trials focused on transplantation are registered on clinicaltrials.gov. Sangamo Therapeutics in 2021 initiated the first trial using CAR-Tregs (NCT04817774), infusing TX200-TR101 into HLA-A02-negative patients awaiting kidney transplants from HLA-A02-positive donors. Encouraging preclinical studies demonstrated specific activation and potent allogeneic Tconv suppression by TX200-TR101, both *in vitro* and *vivo*. No side effects have been reported underscoring its potential as a safe and effective therapeutic alternative (423–425). Quell Therapeutics’s QEL-001, similar to TX200-TR101, utilizes an HLA-A2-specific CAR to abrogate immunosuppression (426, 427)Tregs, and the LIBERATE clinical trial (NCT05234190) is currently enrolling patients who have received HLA A2-mismatched liver transplants 12 months to 5 years prior the time of enrolment.

Sonoma Biotechnologies’s pipeline includes SBT-77-7101, a CAR-Treg product specific to citrullinated vimentin (CV), a known antigen present in the synovial fluid (SF) of Rheumatoid arthritis (RA) patients (428). Preclinical studies (429–432) have shown promise in using Treg therapy for Systemic lupus erythematosus (SLE), demonstrating the as-yet unrealized potential benefit of these novel technologies.

Additionally, an inventive way of using Tregs in autoimmune disease treatment is planning to be introduced into clinical trials on Multiple sclerosis (MS) and type 1 diabetes. The company “Abata Therapeutics” started to engineer Tregs expressing TCRs recognizing tissue-restricted antigens. Similar to the CAR concept, their TCR-based targeted methods reduce systemic suppressive response limiting it to the specific tissue to which autoimmune response is directed. The company PolTREG develops TCR-engineered Tregs in type 1 diabetes. Interestingly, this company works in neuroinflammatory conditions, such as MS and amyotrophic lateral sclerosis, using a CAR-based concept in which CAR-Tregs are designed to strengthen the blood-brain barrier. As the engineered Tregs landscape expands, continued investigation will unveil the most effective strategies to unlock the full potential of engineered Tregs.

## 10 Concluding remarks

Tregs constitute a minor fraction of the broader population of CD4^+^ T-cells. The lack of an exclusive Treg marker together with phenotypical similarities to activated CD4+ effector cells present challenges in the context of cell therapy. However, a variety of markers, often applied in combinatorial approaches identifies functional suppressive Tregs and distinct subsets, and contributed to an enhanced understanding of biological processes. As our knowledge improves and we enter the era of engineered Tregs, significant strides addressing pathological immune-mediated processes can be anticipated. Learnings from ongoing clinical trials, advancement in methods to determine Treg function, together with means to identify patients who are most likely to benefit from Treg therapy, will determine the fate of Treg cell therapy.

## Author contributions

SS: Conceptualization, Writing – original draft, Writing – review & editing, Validation. KK: Writing – original draft, Writing – review & editing, Validation. MG: Writing – original draft, Validation. YH: Writing – review & editing, Validation. DI-G: Writing – original draft, Validation. MP-M: Writing – original draft, Validation. JS: Writing – original draft, Validation. MT: Writing – original draft, Validation. JM: Writing – review & editing, Validation. KL: Writing – review & editing, Validation. NM-T: Supervision, Writing – review & editing, Validation. PT: Supervision, Writing – review & editing, Validation. YO: Supervision, Writing – review & editing. AF: Conceptualization, Funding acquisition, Supervision, Writing – review & editing, Validation.
